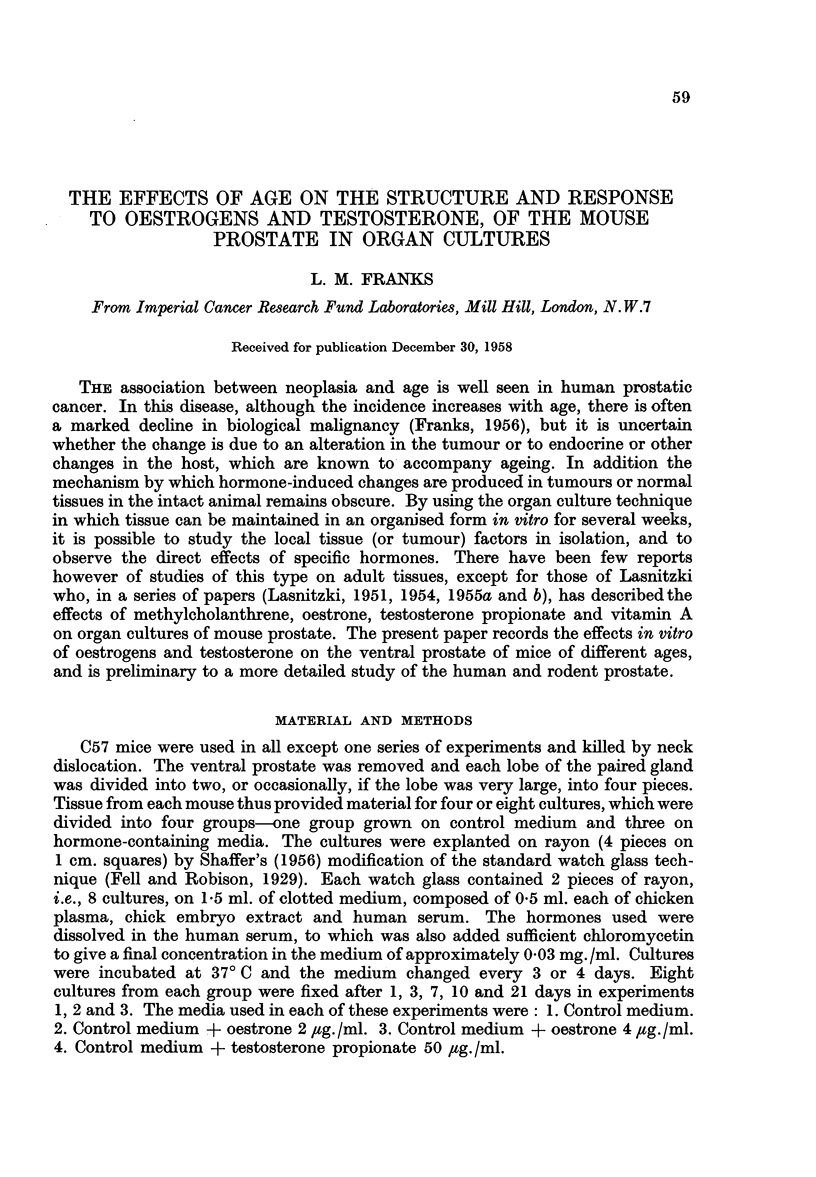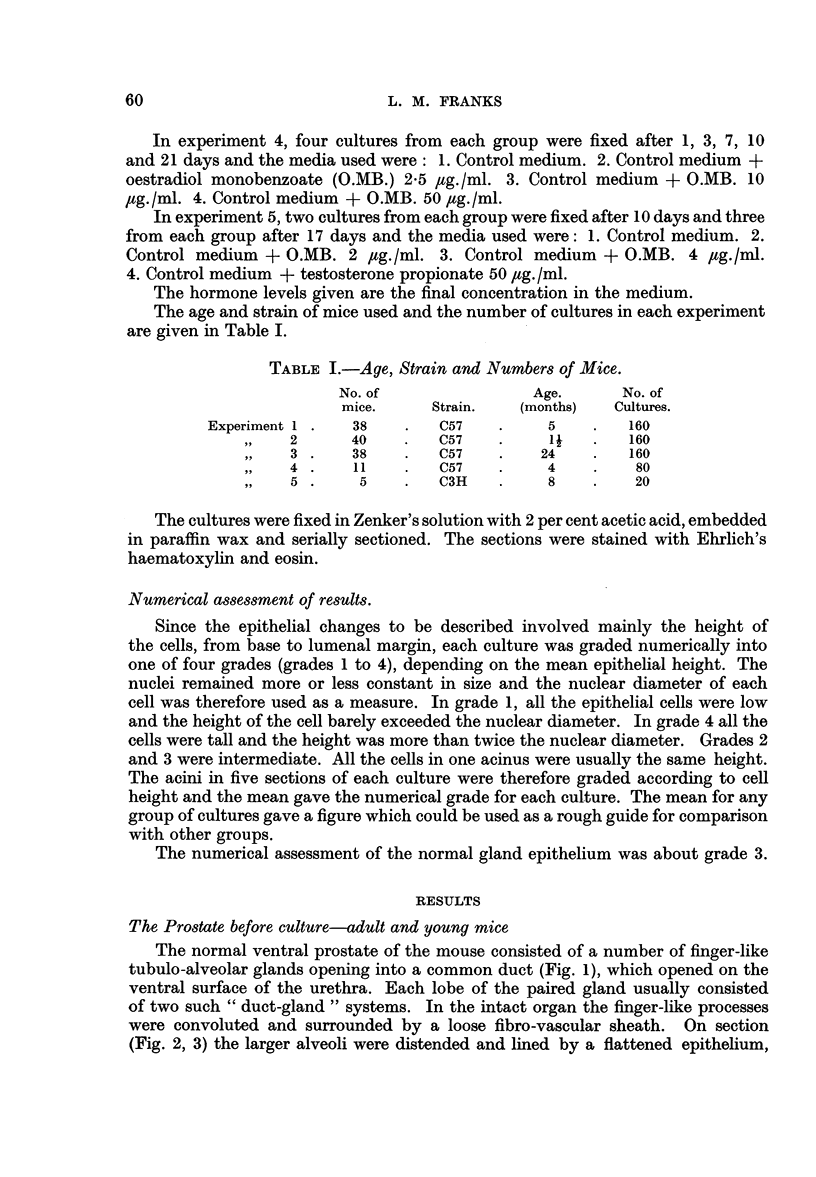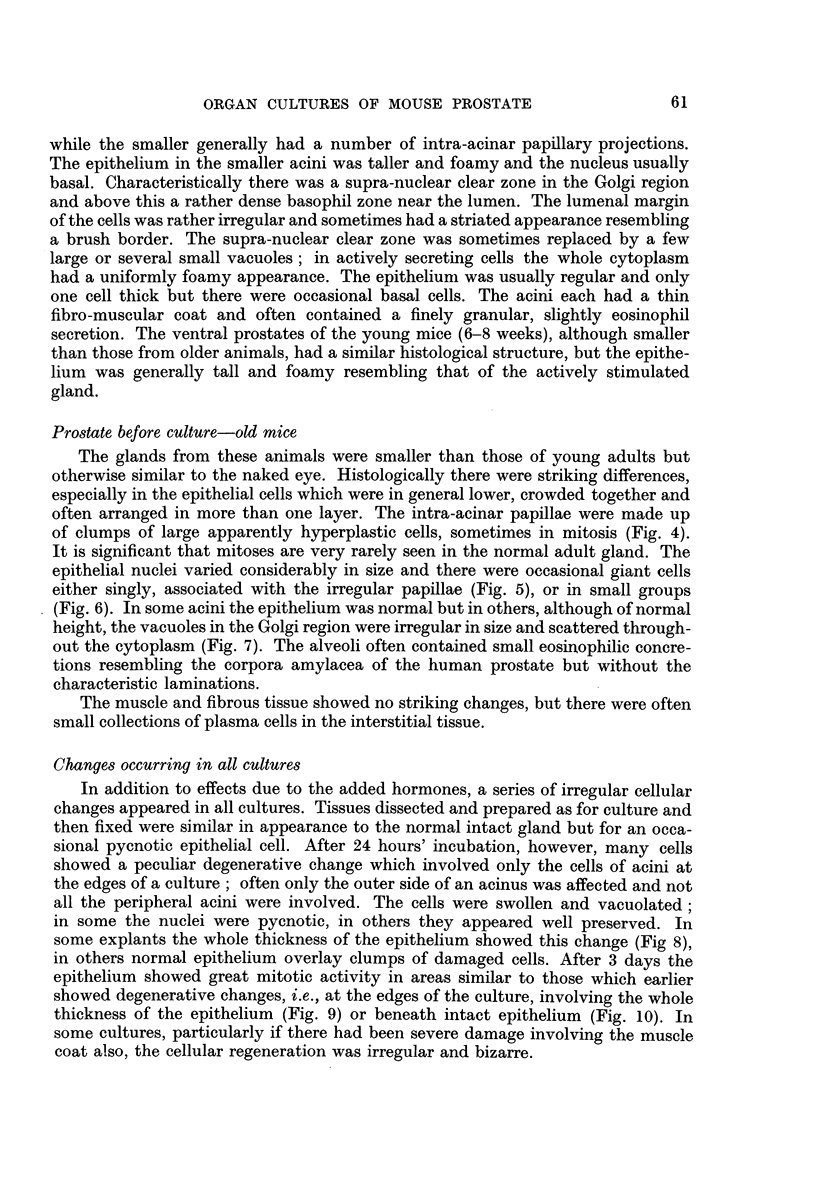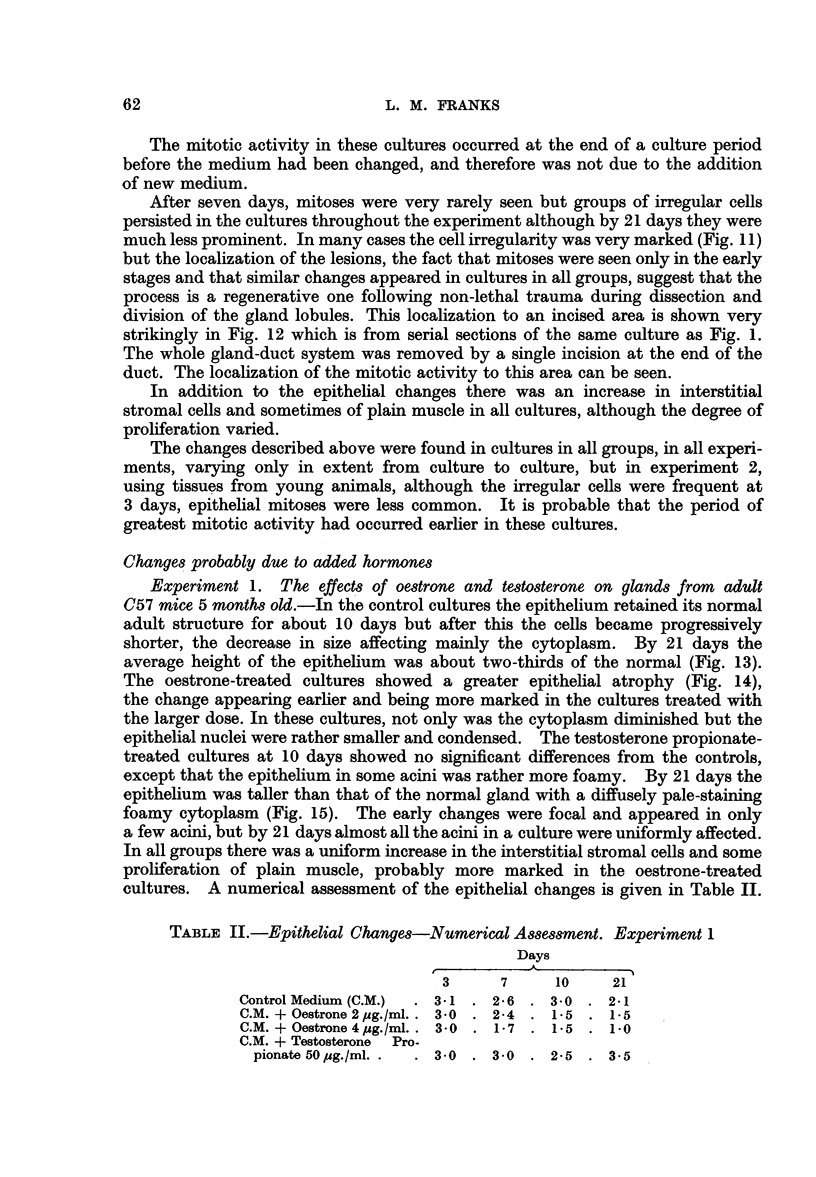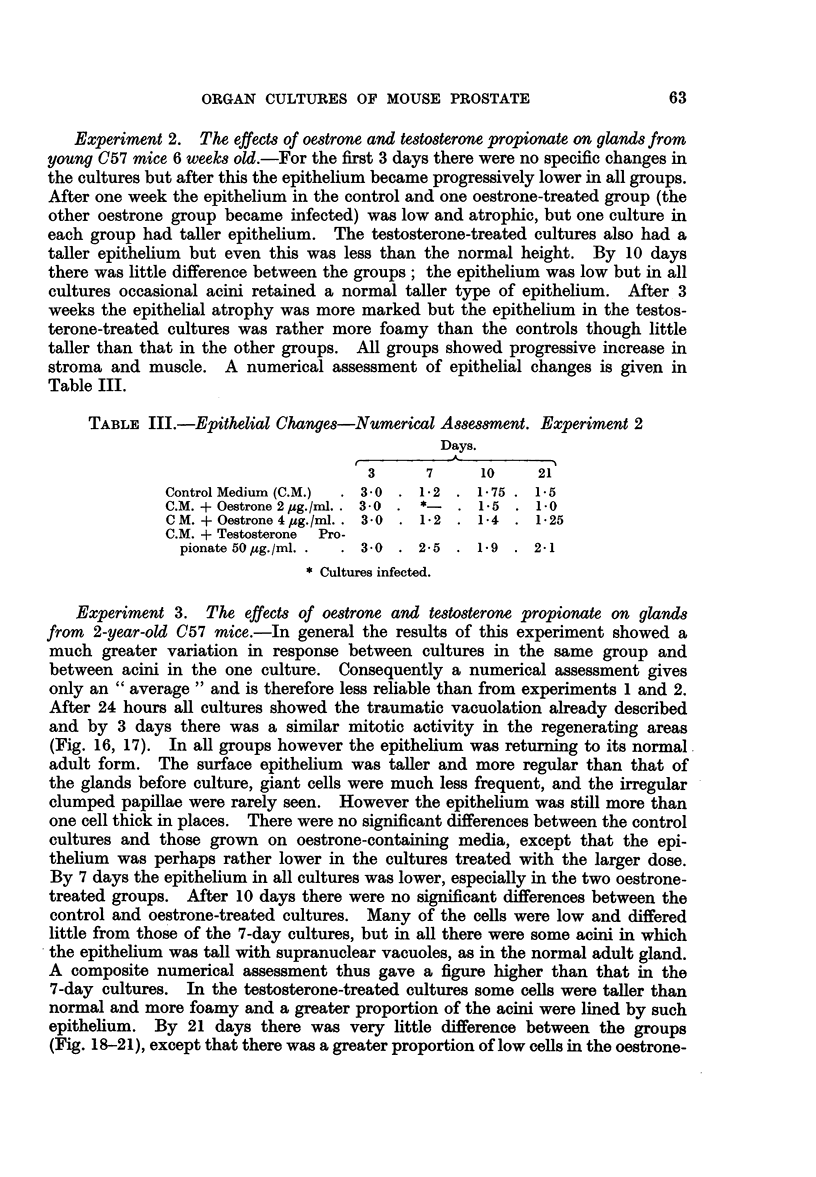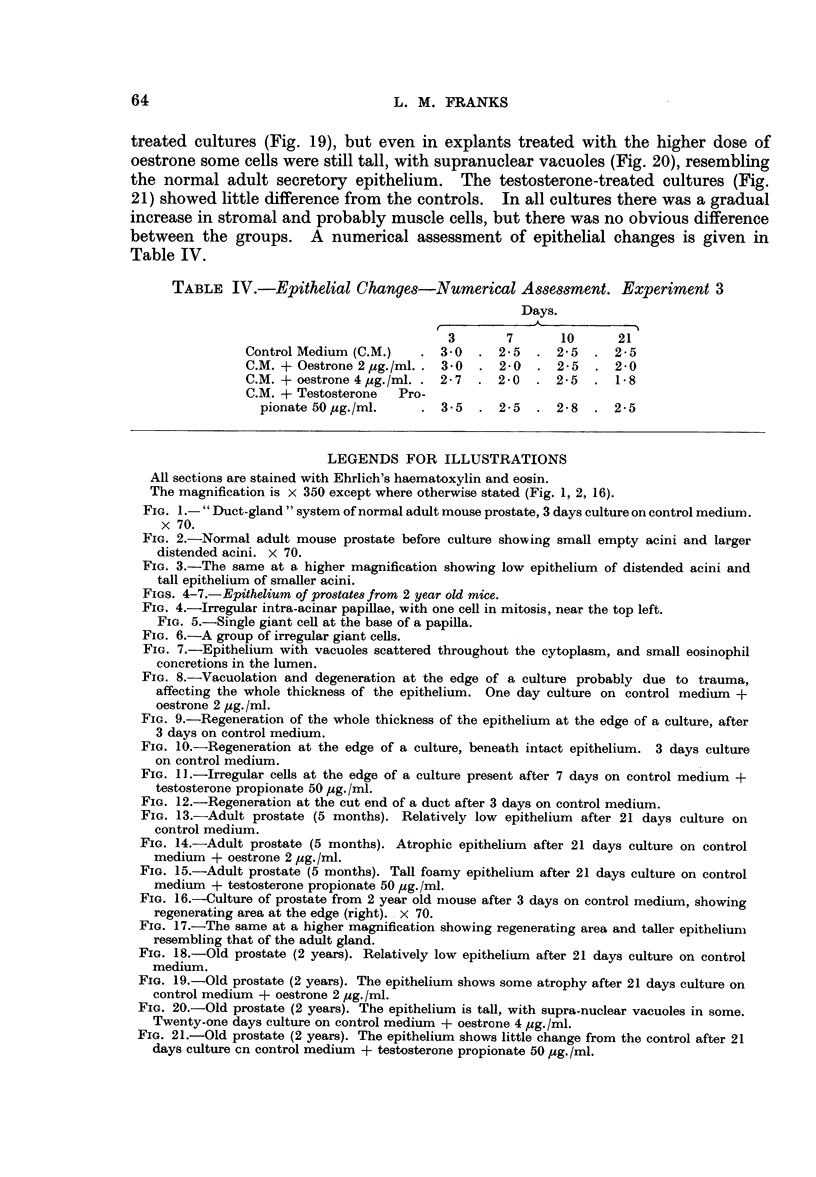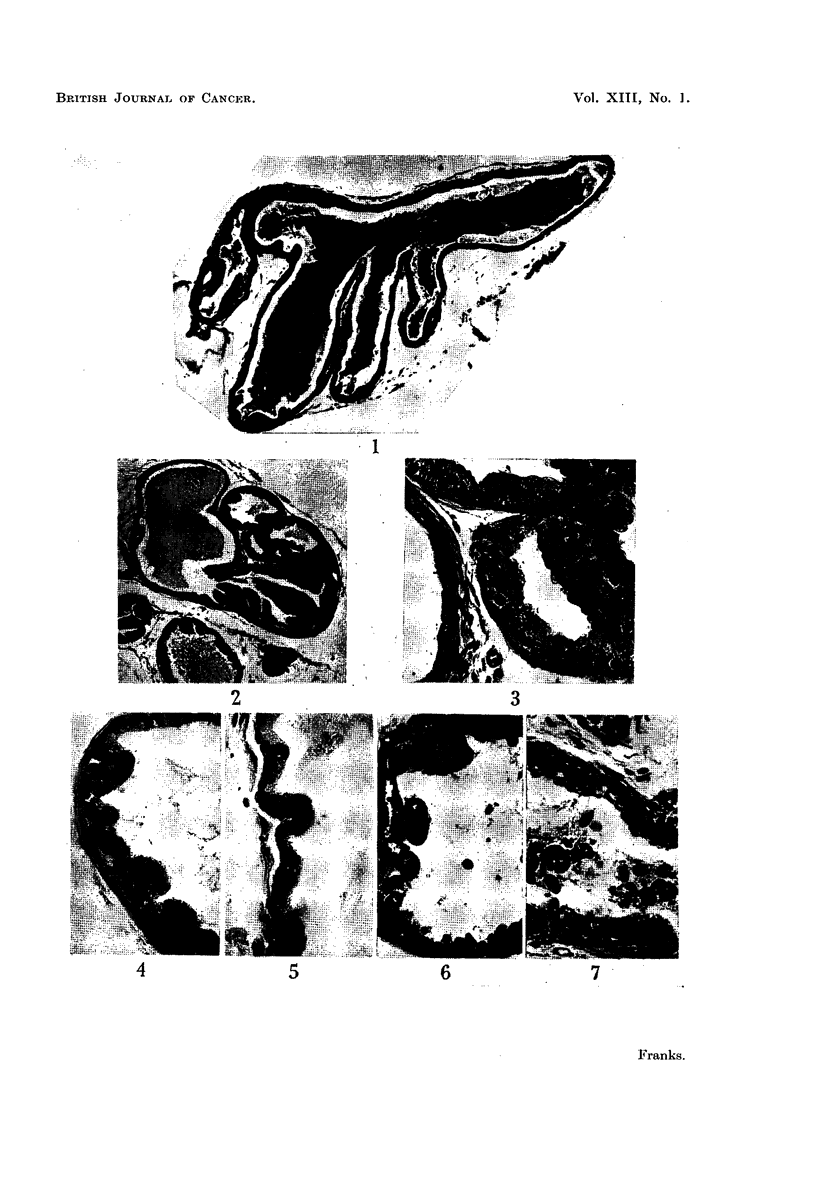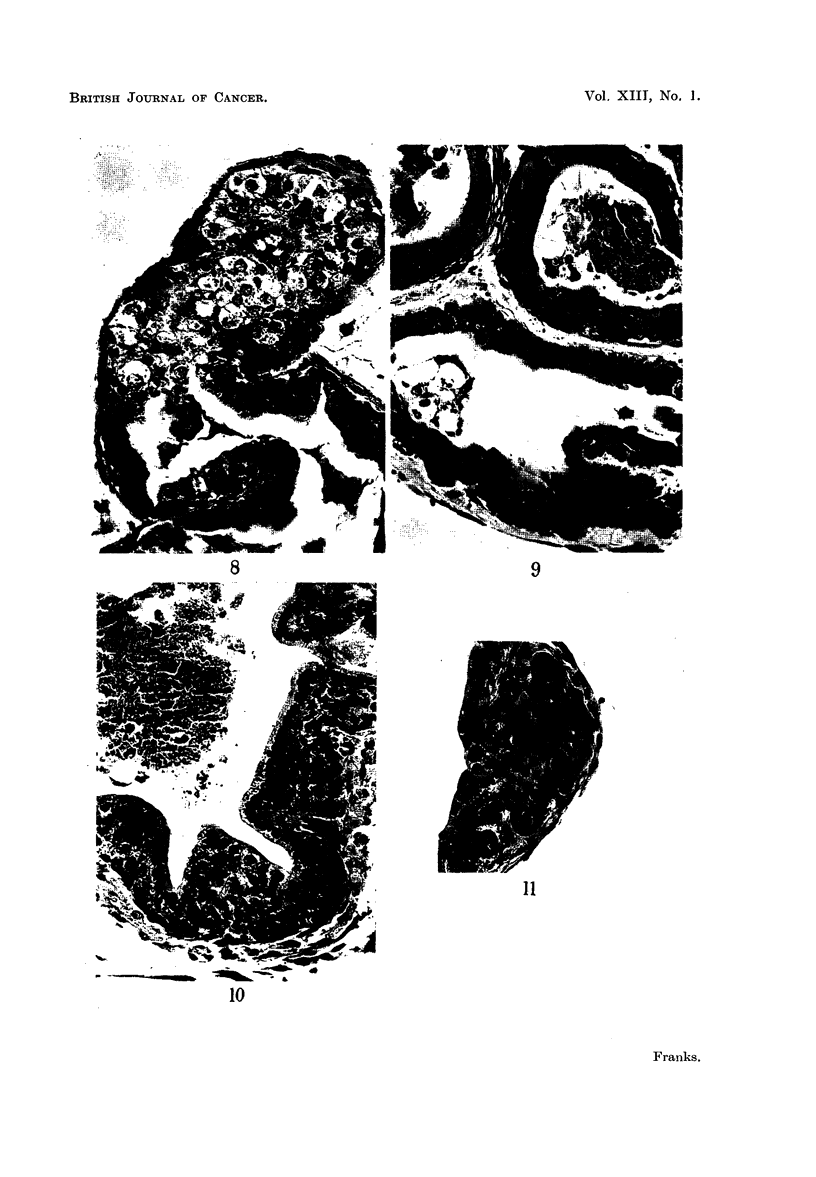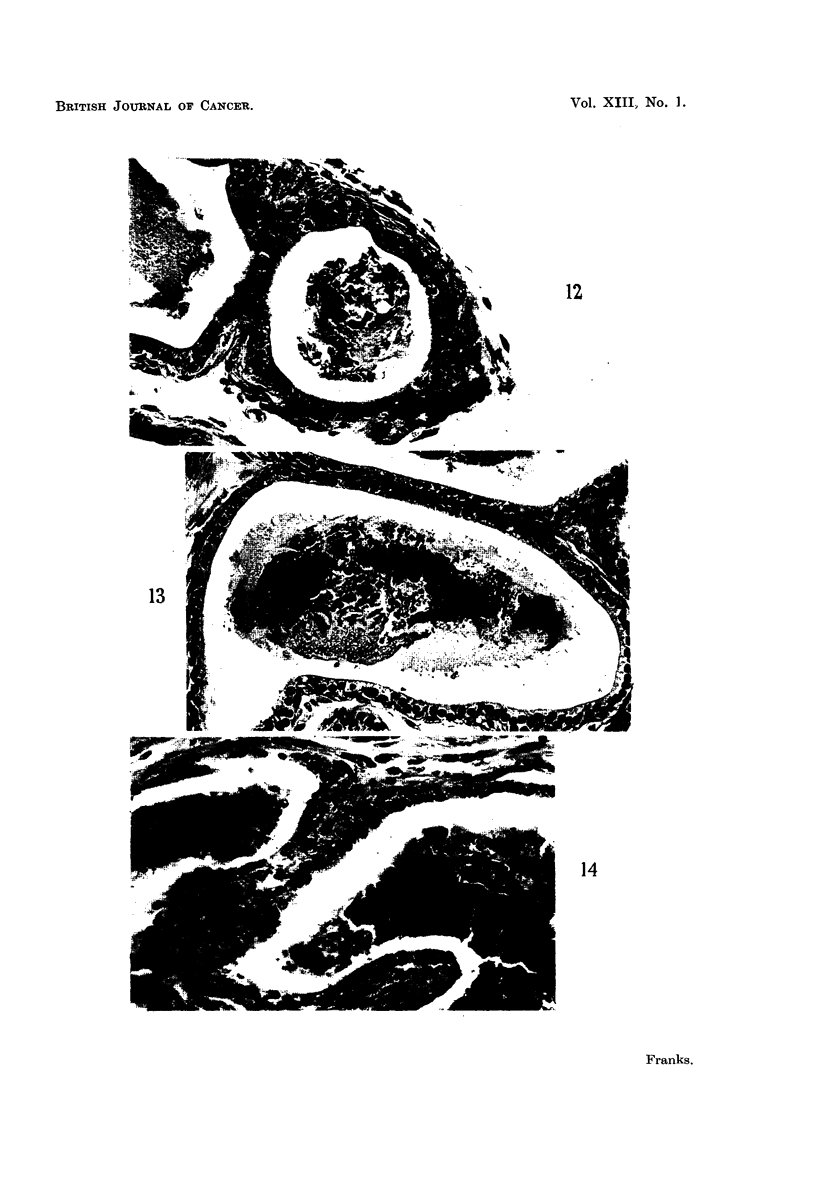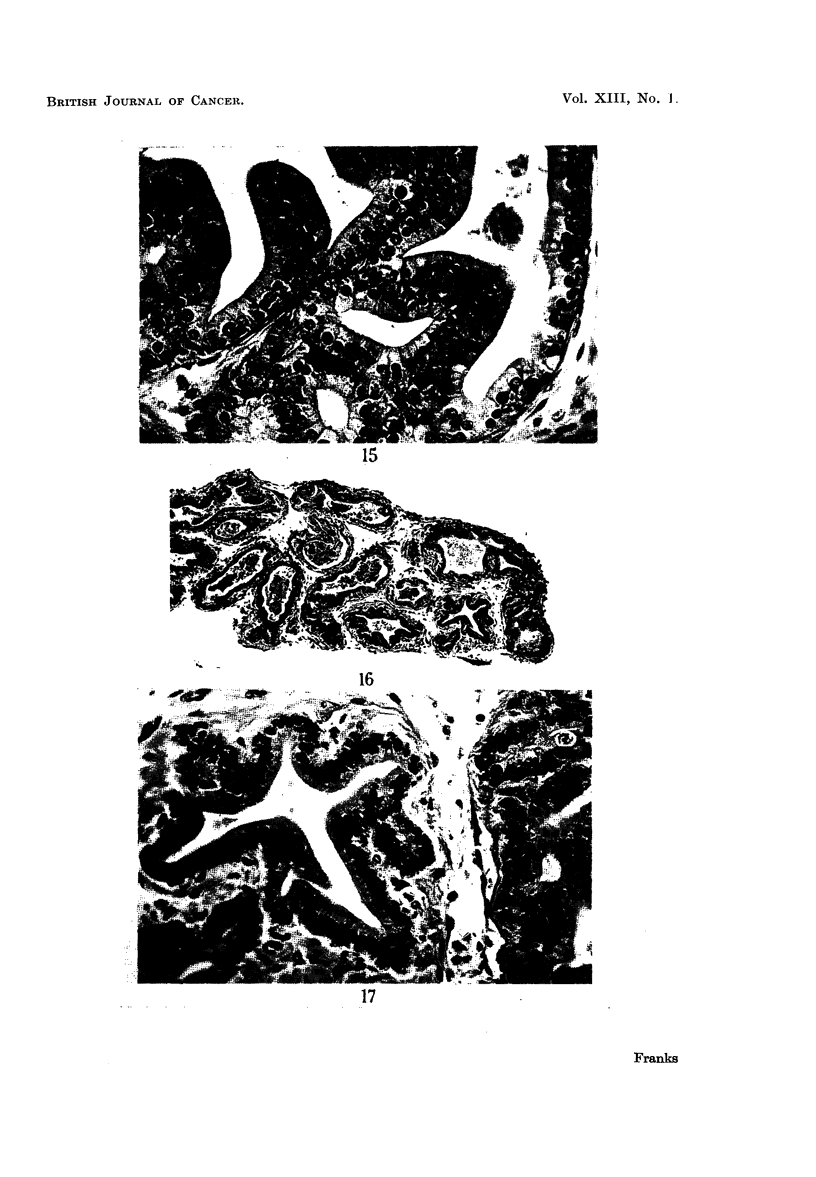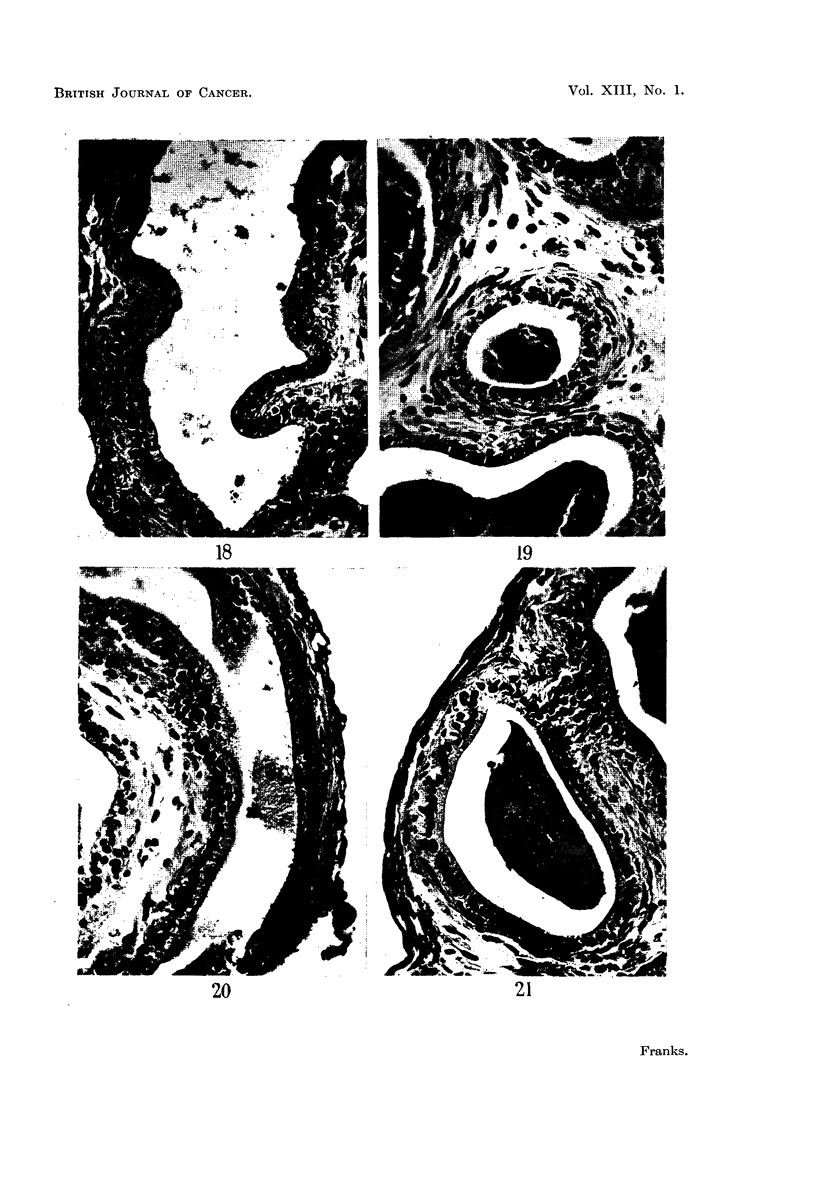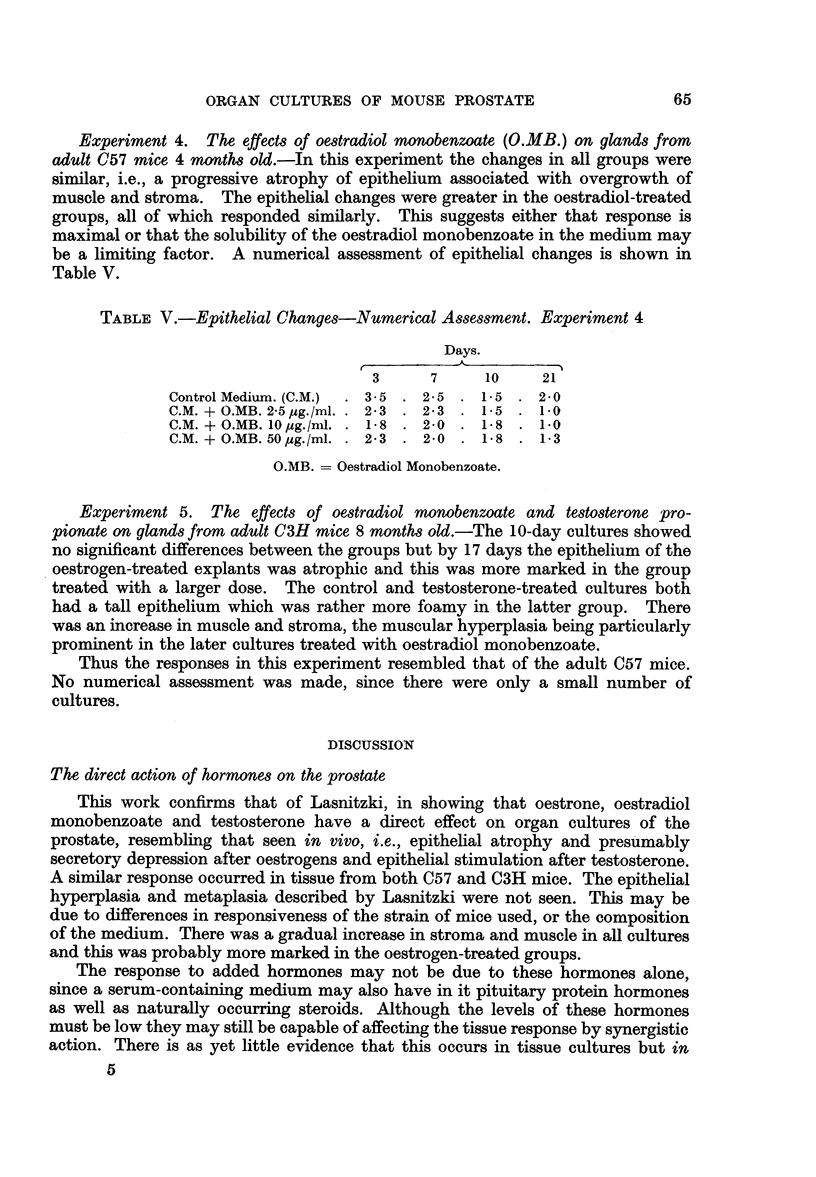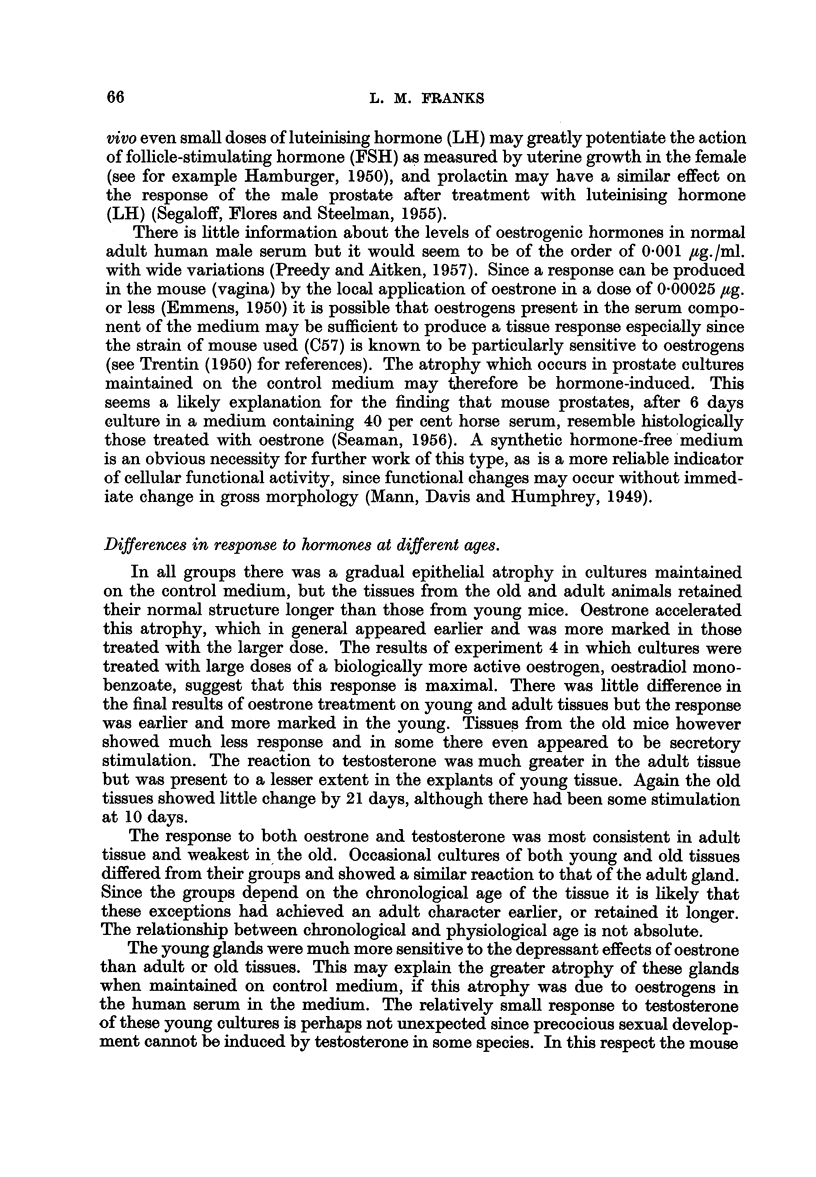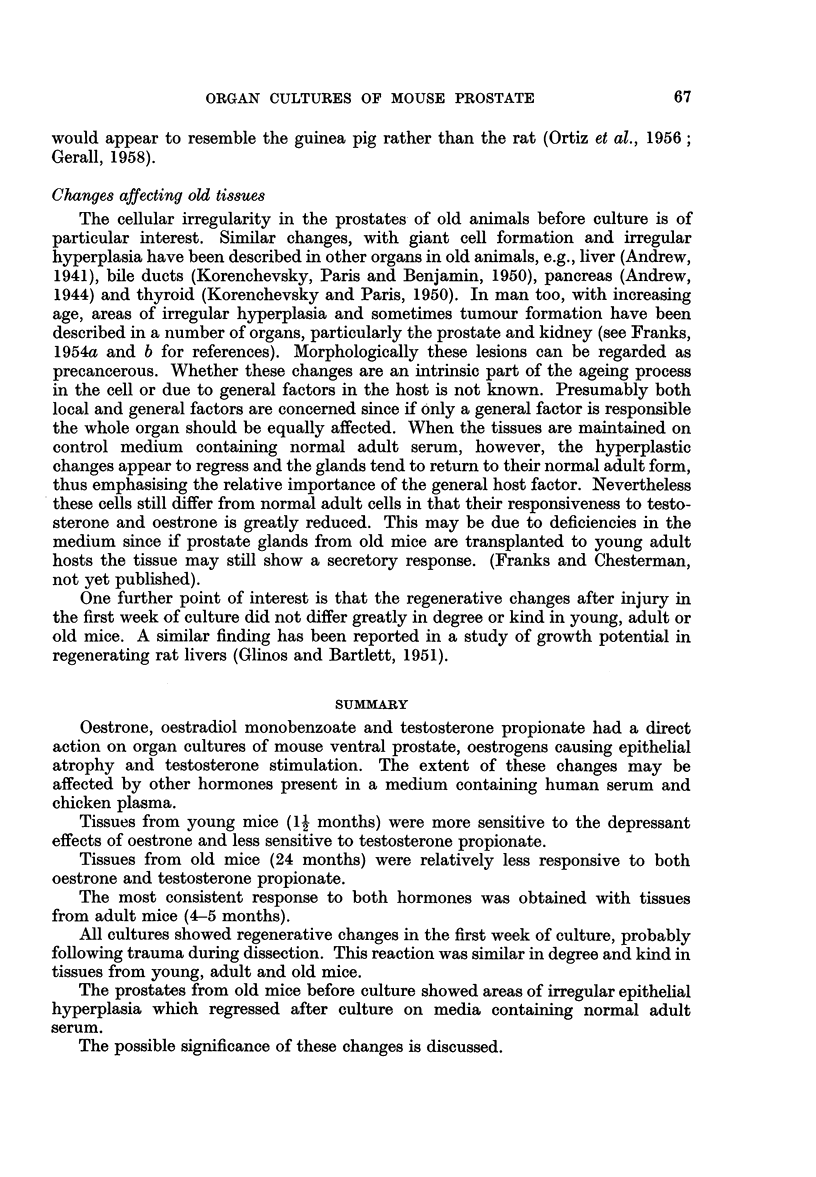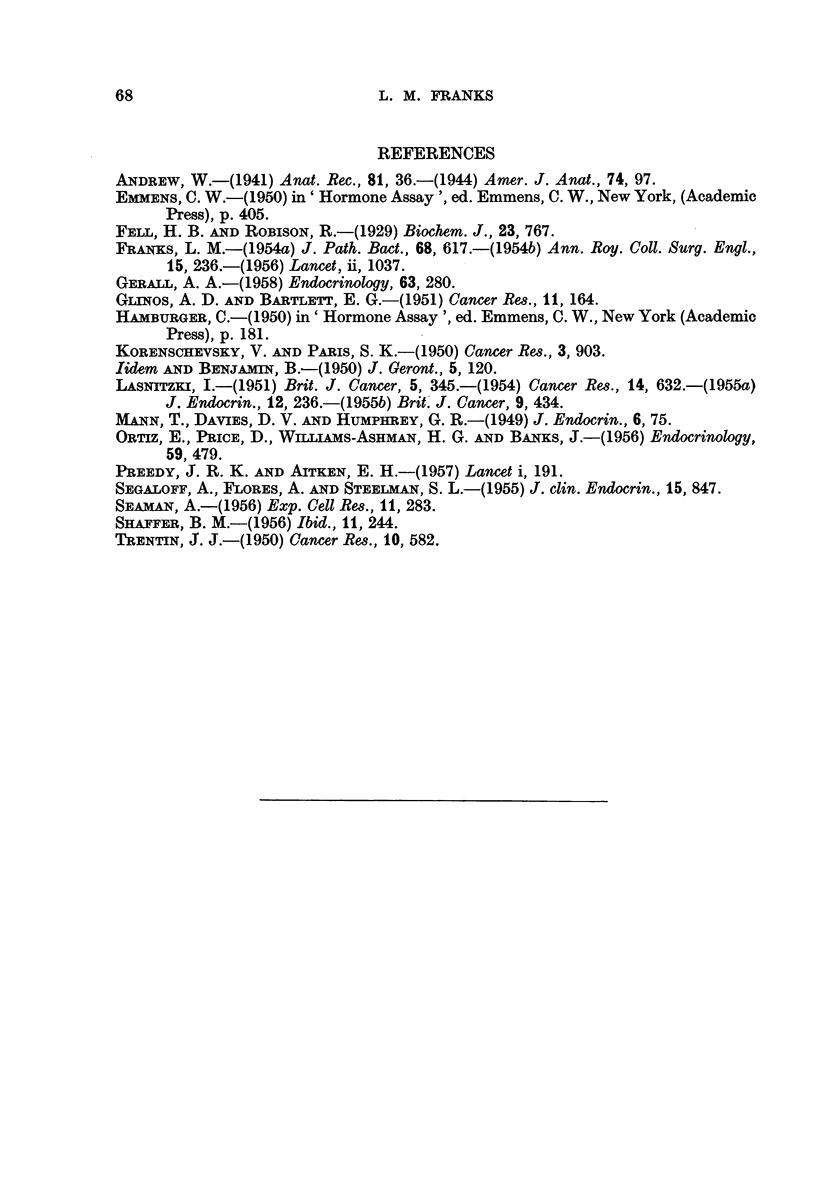# The Effects of Age on the Structure and Response to Oestrogens and Testosterone, of the Mouse Prostate in Organ Cultures

**DOI:** 10.1038/bjc.1959.8

**Published:** 1959-03

**Authors:** L. M. Franks

## Abstract

**Images:**


### THE EFFECTS OF AGE ON THE STRUCTURE AND RESPONSE TO OESTROGENS AND TESTOSTERONE, OF THE MOUSE PROSTATE IN ORGAN CULTURES

L. M. FRANKS

*From Imperial Cancer Research Fund Laboratories, Mill Hill, London, N.W.7*

Received for publication December 30, 1958

THE association between neoplasia and age is well seen in human prostatic cancer. In this disease, although the incidence increases with age, there is often a marked decline in biological malignancy (Franks, 1956), but it is uncertain whether the change is due to an alteration in the tumour or to endocrine or other changes in the host, which are known to accompany ageing. In addition the mechanism by which hormone-induced changes are produced in tumours or normal tissues in the intact animal remains obscure. By using the organ culture technique in which tissue can be maintained in an organised form *in vitro* for several weeks, it is possible to study the local tissue (or tumour) factors in isolation, and to observe the direct effects of specific hormones. There have been few reports however of studies of this type on adult tissues, except for those of Lasnitzki who, in a series of papers (Lasnitzki, 1951, 1954, 1955*a* and *b*), has described the effects of methylcholanthrene, oestrone, testosterone propionate and vitamin A on organ cultures of mouse prostate. The present paper records the effects *in vitro* of oestrogens and testosterone on the ventral prostate of mice of different ages, and is preliminary to a more detailed study of the human and rodent prostate.

### MATERIAL AND METHODS

C57 mice were used in all except one series of experiments and killed by neck dislocation. The ventral prostate was removed and each lobe of the paired gland was divided into two, or occasionally, if the lobe was very large, into four pieces. Tissue from each mouse thus provided material for four or eight cultures, which were divided into four groups—one group grown on control medium and three on hormone-containing media. The cultures were explanted on rayon (4 pieces on 1 cm. squares) by Shaffer's (1956) modification of the standard watch glass technique (Fell and Robison, 1929). Each watch glass contained 2 pieces of rayon, *i.e.*, 8 cultures, on 1·5 ml. of clotted medium, composed of 0·5 ml. each of chicken plasma, chick embryo extract and human serum. The hormones used were dissolved in the human serum, to which was also added sufficient chloromycetin to give a final concentration in the medium of approximately 0·03 mg./ml. Cultures were incubated at 37° C and the medium changed every 3 or 4 days. Eight cultures from each group were fixed after 1, 3, 7, 10 and 21 days in experiments 1, 2 and 3. The media used in each of these experiments were : 1. Control medium. 2. Control medium + oestrone 2 $\mu$g./ml. 3. Control medium + oestrone 4 $\mu$g./ml. 4. Control medium + testosterone propionate 50 $\mu$g./ml.

In experiment 4, four cultures from each group were fixed after 1, 3, 7, 10 and 21 days and the media used were : 1. Control medium. 2. Control medium + oestradiol monobenzoate (O.MB.) 2·5 $\mu$g./ml. 3. Control medium + O.MB. 10 $\mu$g./ml. 4. Control medium + O.MB. 50 $\mu$g./ml.

In experiment 5, two cultures from each group were fixed after 10 days and three from each group after 17 days and the media used were: 1. Control medium. 2. Control medium + O.MB. 2 $\mu$g./ml. 3. Control medium + O.MB. 4 $\mu$g./ml. 4. Control medium + testosterone propionate 50 $\mu$g./ml.

The hormone levels given are the final concentration in the medium.

The age and strain of mice used and the number of cultures in each experiment are given in Table I.

TABLE I.—*Age, Strain and Numbers of Mice.*

| | No. of mice. | Strain. | Age. (months) | No. of Cultures. |
|---|---|---|---|---|
| Experiment 1 | 38 | C57 | 5 | 160 |
| „ 2 | 40 | C57 | 1½ | 160 |
| „ 3 | 38 | C57 | 24 | 160 |
| „ 4 | 11 | C57 | 4 | 80 |
| „ 5 | 5 | C3H | 8 | 20 |

The cultures were fixed in Zenker's solution with 2 per cent acetic acid, embedded in paraffin wax and serially sectioned. The sections were stained with Ehrlich's haematoxylin and eosin.

*Numerical assessment of results.*

Since the epithelial changes to be described involved mainly the height of the cells, from base to lumenal margin, each culture was graded numerically into one of four grades (grades 1 to 4), depending on the mean epithelial height. The nuclei remained more or less constant in size and the nuclear diameter of each cell was therefore used as a measure. In grade 1, all the epithelial cells were low and the height of the cell barely exceeded the nuclear diameter. In grade 4 all the cells were tall and the height was more than twice the nuclear diameter. Grades 2 and 3 were intermediate. All the cells in one acinus were usually the same height. The acini in five sections of each culture were therefore graded according to cell height and the mean gave the numerical grade for each culture. The mean for any group of cultures gave a figure which could be used as a rough guide for comparison with other groups.

The numerical assessment of the normal gland epithelium was about grade 3.

### RESULTS

*The Prostate before culture—adult and young mice*

The normal ventral prostate of the mouse consisted of a number of finger-like tubulo-alveolar glands opening into a common duct (Fig. 1), which opened on the ventral surface of the urethra. Each lobe of the paired gland usually consisted of two such " duct-gland " systems. In the intact organ the finger-like processes were convoluted and surrounded by a loose fibro-vascular sheath. On section (Fig. 2, 3) the larger alveoli were distended and lined by a flattened epithelium, while the smaller generally had a number of intra-acinar papillary projections. The epithelium in the smaller acini was taller and foamy and the nucleus usually basal. Characteristically there was a supra-nuclear clear zone in the Golgi region and above this a rather dense basophil zone near the lumen. The lumenal margin of the cells was rather irregular and sometimes had a striated appearance resembling a brush border. The supra-nuclear clear zone was sometimes replaced by a few large or several small vacuoles ; in actively secreting cells the whole cytoplasm had a uniformly foamy appearance. The epithelium was usually regular and only one cell thick but there were occasional basal cells. The acini each had a thin fibro-muscular coat and often contained a finely granular, slightly eosinophil secretion. The ventral prostates of the young mice (6–8 weeks), although smaller than those from older animals, had a similar histological structure, but the epithelium was generally tall and foamy resembling that of the actively stimulated gland.

*Prostate before culture—old mice*

The glands from these animals were smaller than those of young adults but otherwise similar to the naked eye. Histologically there were striking differences, especially in the epithelial cells which were in general lower, crowded together and often arranged in more than one layer. The intra-acinar papillae were made up of clumps of large apparently hyperplastic cells, sometimes in mitosis (Fig. 4). It is significant that mitoses are very rarely seen in the normal adult gland. The epithelial nuclei varied considerably in size and there were occasional giant cells either singly, associated with the irregular papillae (Fig. 5), or in small groups (Fig. 6). In some acini the epithelium was normal but in others, although of normal height, the vacuoles in the Golgi region were irregular in size and scattered throughout the cytoplasm (Fig. 7). The alveoli often contained small eosinophilic concretions resembling the corpora amylacea of the human prostate but without the characteristic laminations.

The muscle and fibrous tissue showed no striking changes, but there were often small collections of plasma cells in the interstitial tissue.

*Changes occurring in all cultures*

In addition to effects due to the added hormones, a series of irregular cellular changes appeared in all cultures. Tissues dissected and prepared as for culture and then fixed were similar in appearance to the normal intact gland but for an occasional pycnotic epithelial cell. After 24 hours' incubation, however, many cells showed a peculiar degenerative change which involved only the cells of acini at the edges of a culture ; often only the outer side of an acinus was affected and not all the peripheral acini were involved. The cells were swollen and vacuolated ; in some the nuclei were pycnotic, in others they appeared well preserved. In some explants the whole thickness of the epithelium showed this change (Fig 8), in others normal epithelium overlay clumps of damaged cells. After 3 days the epithelium showed great mitotic activity in areas similar to those which earlier showed degenerative changes, *i.e.*, at the edges of the culture, involving the whole thickness of the epithelium (Fig. 9) or beneath intact epithelium (Fig. 10). In some cultures, particularly if there had been severe damage involving the muscle coat also, the cellular regeneration was irregular and bizarre.

The mitotic activity in these cultures occurred at the end of a culture period before the medium had been changed, and therefore was not due to the addition of new medium.

After seven days, mitoses were very rarely seen but groups of irregular cells persisted in the cultures throughout the experiment although by 21 days they were much less prominent. In many cases the cell irregularity was very marked (Fig. 11) but the localization of the lesions, the fact that mitoses were seen only in the early stages and that similar changes appeared in cultures in all groups, suggest that the process is a regenerative one following non-lethal trauma during dissection and division of the gland lobules. This localization to an incised area is shown very strikingly in Fig. 12 which is from serial sections of the same culture as Fig. 1. The whole gland-duct system was removed by a single incision at the end of the duct. The localization of the mitotic activity to this area can be seen.

In addition to the epithelial changes there was an increase in interstitial stromal cells and sometimes of plain muscle in all cultures, although the degree of proliferation varied.

The changes described above were found in cultures in all groups, in all experiments, varying only in extent from culture to culture, but in experiment 2, using tissues from young animals, although the irregular cells were frequent at 3 days, epithelial mitoses were less common. It is probable that the period of greatest mitotic activity had occurred earlier in these cultures.

*Changes probably due to added hormones*

*Experiment* 1. *The effects of oestrone and testosterone on glands from adult C*57 *mice* 5 *months old.*—In the control cultures the epithelium retained its normal adult structure for about 10 days but after this the cells became progressively shorter, the decrease in size affecting mainly the cytoplasm. By 21 days the average height of the epithelium was about two-thirds of the normal (Fig. 13). The oestrone-treated cultures showed a greater epithelial atrophy (Fig. 14), the change appearing earlier and being more marked in the cultures treated with the larger dose. In these cultures, not only was the cytoplasm diminished but the epithelial nuclei were rather smaller and condensed. The testosterone propionate-treated cultures at 10 days showed no significant differences from the controls, except that the epithelium in some acini was rather more foamy. By 21 days the epithelium was taller than that of the normal gland with a diffusely pale-staining foamy cytoplasm (Fig. 15). The early changes were focal and appeared in only a few acini, but by 21 days almost all the acini in a culture were uniformly affected. In all groups there was a uniform increase in the interstitial stromal cells and some proliferation of plain muscle, probably more marked in the oestrone-treated cultures. A numerical assessment of the epithelial changes is given in Table II.

TABLE II.—*Epithelial Changes—Numerical Assessment. Experiment* 1

| | Days | | | |
|---|---|---|---|---|
| | 3 | 7 | 10 | 21 |
| Control Medium (C.M.) | 3·1 | 2·6 | 3·0 | 2·1 |
| C.M. + Oestrone 2 μg./ml. | 3·0 | 2·4 | 1·5 | 1·5 |
| C.M. + Oestrone 4 μg./ml. | 3·0 | 1·7 | 1·5 | 1·0 |
| C.M. + Testosterone Propionate 50 μg./ml. | 3·0 | 3·0 | 2·5 | 3·5 |

*Experiment* 2. *The effects of oestrone and testosterone propionate on glands from young C57 mice 6 weeks old.*—For the first 3 days there were no specific changes in the cultures but after this the epithelium became progressively lower in all groups. After one week the epithelium in the control and one oestrone-treated group (the other oestrone group became infected) was low and atrophic, but one culture in each group had taller epithelium. The testosterone-treated cultures also had a taller epithelium but even this was less than the normal height. By 10 days there was little difference between the groups ; the epithelium was low but in all cultures occasional acini retained a normal taller type of epithelium. After 3 weeks the epithelial atrophy was more marked but the epithelium in the testosterone-treated cultures was rather more foamy than the controls though little taller than that in the other groups. All groups showed progressive increase in stroma and muscle. A numerical assessment of epithelial changes is given in Table III.

TABLE III.—*Epithelial Changes—Numerical Assessment. Experiment* 2

| | Days. | | | |
|---|---|---|---|---|
| | 3 | 7 | 10 | 21 |
| Control Medium (C.M.) | 3·0 | 1·2 | 1·75 | 1·5 |
| C.M. + Oestrone 2 μg./ml. | 3·0 | *— | 1·5 | 1·0 |
| C M. + Oestrone 4 μg./ml. | 3·0 | 1·2 | 1·4 | 1·25 |
| C.M. + Testosterone Propionate 50 μg./ml. | 3·0 | 2·5 | 1·9 | 2·1 |

* Cultures infected.

*Experiment* 3. *The effects of oestrone and testosterone propionate on glands from 2-year-old C57 mice.*—In general the results of this experiment showed a much greater variation in response between cultures in the same group and between acini in the one culture. Consequently a numerical assessment gives only an " average " and is therefore less reliable than from experiments 1 and 2. After 24 hours all cultures showed the traumatic vacuolation already described and by 3 days there was a similar mitotic activity in the regenerating areas (Fig. 16, 17). In all groups however the epithelium was returning to its normal adult form. The surface epithelium was taller and more regular than that of the glands before culture, giant cells were much less frequent, and the irregular clumped papillae were rarely seen. However the epithelium was still more than one cell thick in places. There were no significant differences between the control cultures and those grown on oestrone-containing media, except that the epithelium was perhaps rather lower in the cultures treated with the larger dose. By 7 days the epithelium in all cultures was lower, especially in the two oestrone-treated groups. After 10 days there were no significant differences between the control and oestrone-treated cultures. Many of the cells were low and differed little from those of the 7-day cultures, but in all there were some acini in which the epithelium was tall with supranuclear vacuoles, as in the normal adult gland. A composite numerical assessment thus gave a figure higher than that in the 7-day cultures. In the testosterone-treated cultures some cells were taller than normal and more foamy and a greater proportion of the acini were lined by such epithelium. By 21 days there was very little difference between the groups (Fig. 18–21), except that there was a greater proportion of low cells in the oestronetreated cultures (Fig. 19), but even in explants treated with the higher dose of oestrone some cells were still tall, with supranuclear vacuoles (Fig. 20), resembling the normal adult secretory epithelium. The testosterone-treated cultures (Fig. 21) showed little difference from the controls. In all cultures there was a gradual increase in stromal and probably muscle cells, but there was no obvious difference between the groups. A numerical assessment of epithelial changes is given in Table IV.

TABLE IV.—*Epithelial Changes—Numerical Assessment. Experiment* 3

| | Days. | | | |
|---|---|---|---|---|
| | 3 | 7 | 10 | 21 |
| Control Medium (C.M.) | 3·0 | 2·5 | 2·5 | 2·5 |
| C.M. + Oestrone 2 μg./ml. | 3·0 | 2·0 | 2·5 | 2·0 |
| C.M. + oestrone 4 μg./ml. | 2·7 | 2·0 | 2·5 | 1·8 |
| C.M. + Testosterone Propionate 50 μg./ml. | 3·5 | 2·5 | 2·8 | 2·5 |

### LEGENDS FOR ILLUSTRATIONS

All sections are stained with Ehrlich's haematoxylin and eosin.

The magnification is × 350 except where otherwise stated (Fig. 1, 2, 16).

FIG. 1.—" Duct-gland " system of normal adult mouse prostate, 3 days culture on control medium. × 70.

FIG. 2.—Normal adult mouse prostate before culture showing small empty acini and larger distended acini. × 70.

FIG. 3.—The same at a higher magnification showing low epithelium of distended acini and tall epithelium of smaller acini.

FIGS. 4–7.—*Epithelium of prostates from* 2 *year old mice.*

FIG. 4.—Irregular intra-acinar papillae, with one cell in mitosis, near the top left.

FIG. 5.—Single giant cell at the base of a papilla.

FIG. 6.—A group of irregular giant cells.

FIG. 7.—Epithelium with vacuoles scattered throughout the cytoplasm, and small eosinophil concretions in the lumen.

FIG. 8.—Vacuolation and degeneration at the edge of a culture probably due to trauma, affecting the whole thickness of the epithelium. One day culture on control medium + oestrone 2 μg./ml.

FIG. 9.—Regeneration of the whole thickness of the epithelium at the edge of a culture, after 3 days on control medium.

FIG. 10.—Regeneration at the edge of a culture, beneath intact epithelium. 3 days culture on control medium.

FIG. 11.—Irregular cells at the edge of a culture present after 7 days on control medium + testosterone propionate 50 μg./ml.

FIG. 12.—Regeneration at the cut end of a duct after 3 days on control medium.

FIG. 13.—Adult prostate (5 months). Relatively low epithelium after 21 days culture on control medium.

FIG. 14.—Adult prostate (5 months). Atrophic epithelium after 21 days culture on control medium + oestrone 2 μg./ml.

FIG. 15.—Adult prostate (5 months). Tall foamy epithelium after 21 days culture on control medium + testosterone propionate 50 μg./ml.

FIG. 16.—Culture of prostate from 2 year old mouse after 3 days on control medium, showing regenerating area at the edge (right). × 70.

FIG. 17.—The same at a higher magnification showing regenerating area and taller epithelium resembling that of the adult gland.

FIG. 18.—Old prostate (2 years). Relatively low epithelium after 21 days culture on control medium.

FIG. 19.—Old prostate (2 years). The epithelium shows some atrophy after 21 days culture on control medium + oestrone 2 μg./ml.

FIG. 20.—Old prostate (2 years). The epithelium is tall, with supra-nuclear vacuoles in some. Twenty-one days culture on control medium + oestrone 4 μg./ml.

FIG. 21.—Old prostate (2 years). The epithelium shows little change from the control after 21 days culture cn control medium + testosterone propionate 50 μg./ml.

1

2

3

4

5

6

7

Franks.

8

9

10

11

12

13

14

Franks.

15

16

17

18

19

20

21

Franks.

*Experiment* 4. *The effects of oestradiol monobenzoate* (*O.MB.*) *on glands from adult C*57 *mice* 4 *months old.*—In this experiment the changes in all groups were similar, i.e., a progressive atrophy of epithelium associated with overgrowth of muscle and stroma. The epithelial changes were greater in the oestradiol-treated groups, all of which responded similarly. This suggests either that response is maximal or that the solubility of the oestradiol monobenzoate in the medium may be a limiting factor. A numerical assessment of epithelial changes is shown in Table V.

TABLE V.—*Epithelial Changes—Numerical Assessment. Experiment* 4

| | Days. | | | |
|---|---|---|---|---|
| | 3 | 7 | 10 | 21 |
| Control Medium. (C.M.) | 3·5 | 2·5 | 1·5 | 2·0 |
| C.M. + O.MB. 2·5 μg./ml. | 2·3 | 2·3 | 1·5 | 1·0 |
| C.M. + O.MB. 10 μg./ml. | 1·8 | 2·0 | 1·8 | 1·0 |
| C.M. + O.MB. 50 μg./ml. | 2·3 | 2·0 | 1·8 | 1·3 |

O.MB. = Oestradiol Monobenzoate.

*Experiment* 5. *The effects of oestradiol monobenzoate and testosterone propionate on glands from adult C3H mice* 8 *months old.*—The 10-day cultures showed no significant differences between the groups but by 17 days the epithelium of the oestrogen-treated explants was atrophic and this was more marked in the group treated with a larger dose. The control and testosterone-treated cultures both had a tall epithelium which was rather more foamy in the latter group. There was an increase in muscle and stroma, the muscular hyperplasia being particularly prominent in the later cultures treated with oestradiol monobenzoate.

Thus the responses in this experiment resembled that of the adult C57 mice. No numerical assessment was made, since there were only a small number of cultures.

### DISCUSSION

### *The direct action of hormones on the prostate*

This work confirms that of Lasnitzki, in showing that oestrone, oestradiol monobenzoate and testosterone have a direct effect on organ cultures of the prostate, resembling that seen *in vivo*, *i.e.*, epithelial atrophy and presumably secretory depression after oestrogens and epithelial stimulation after testosterone. A similar response occurred in tissue from both C57 and C3H mice. The epithelial hyperplasia and metaplasia described by Lasnitzki were not seen. This may be due to differences in responsiveness of the strain of mice used, or the composition of the medium. There was a gradual increase in stroma and muscle in all cultures and this was probably more marked in the oestrogen-treated groups.

The response to added hormones may not be due to these hormones alone, since a serum-containing medium may also have in it pituitary protein hormones as well as naturally occurring steroids. Although the levels of these hormones must be low they may still be capable of affecting the tissue response by synergistic action. There is as yet little evidence that this occurs in tissue cultures but *in*

*vivo* even small doses of luteinising hormone (LH) may greatly potentiate the action of follicle-stimulating hormone (FSH) as measured by uterine growth in the female (see for example Hamburger, 1950), and prolactin may have a similar effect on the response of the male prostate after treatment with luteinising hormone (LH) (Segaloff, Flores and Steelman, 1955).

There is little information about the levels of oestrogenic hormones in normal adult human male serum but it would seem to be of the order of 0·001 $\mu$g./ml. with wide variations (Preedy and Aitken, 1957). Since a response can be produced in the mouse (vagina) by the local application of oestrone in a dose of 0·00025 $\mu$g. or less (Emmens, 1950) it is possible that oestrogens present in the serum component of the medium may be sufficient to produce a tissue response especially since the strain of mouse used (C57) is known to be particularly sensitive to oestrogens (see Trentin (1950) for references). The atrophy which occurs in prostate cultures maintained on the control medium may therefore be hormone-induced. This seems a likely explanation for the finding that mouse prostates, after 6 days culture in a medium containing 40 per cent horse serum, resemble histologically those treated with oestrone (Seaman, 1956). A synthetic hormone-free medium is an obvious necessity for further work of this type, as is a more reliable indicator of cellular functional activity, since functional changes may occur without immediate change in gross morphology (Mann, Davis and Humphrey, 1949).

*Differences in response to hormones at different ages.*

In all groups there was a gradual epithelial atrophy in cultures maintained on the control medium, but the tissues from the old and adult animals retained their normal structure longer than those from young mice. Oestrone accelerated this atrophy, which in general appeared earlier and was more marked in those treated with the larger dose. The results of experiment 4 in which cultures were treated with large doses of a biologically more active oestrogen, oestradiol monobenzoate, suggest that this response is maximal. There was little difference in the final results of oestrone treatment on young and adult tissues but the response was earlier and more marked in the young. Tissues from the old mice however showed much less response and in some there even appeared to be secretory stimulation. The reaction to testosterone was much greater in the adult tissue but was present to a lesser extent in the explants of young tissue. Again the old tissues showed little change by 21 days, although there had been some stimulation at 10 days.

The response to both oestrone and testosterone was most consistent in adult tissue and weakest in the old. Occasional cultures of both young and old tissues differed from their groups and showed a similar reaction to that of the adult gland. Since the groups depend on the chronological age of the tissue it is likely that these exceptions had achieved an adult character earlier, or retained it longer. The relationship between chronological and physiological age is not absolute.

The young glands were much more sensitive to the depressant effects of oestrone than adult or old tissues. This may explain the greater atrophy of these glands when maintained on control medium, if this atrophy was due to oestrogens in the human serum in the medium. The relatively small response to testosterone of these young cultures is perhaps not unexpected since precocious sexual development cannot be induced by testosterone in some species. In this respect the mouse would appear to resemble the guinea pig rather than the rat (Ortiz *et al.*, 1956; Gerall, 1958).

*Changes affecting old tissues*

The cellular irregularity in the prostates of old animals before culture is of particular interest. Similar changes, with giant cell formation and irregular hyperplasia have been described in other organs in old animals, e.g., liver (Andrew, 1941), bile ducts (Korenchevsky, Paris and Benjamin, 1950), pancreas (Andrew, 1944) and thyroid (Korenchevsky and Paris, 1950). In man too, with increasing age, areas of irregular hyperplasia and sometimes tumour formation have been described in a number of organs, particularly the prostate and kidney (see Franks, 1954*a* and *b* for references). Morphologically these lesions can be regarded as precancerous. Whether these changes are an intrinsic part of the ageing process in the cell or due to general factors in the host is not known. Presumably both local and general factors are concerned since if only a general factor is responsible the whole organ should be equally affected. When the tissues are maintained on control medium containing normal adult serum, however, the hyperplastic changes appear to regress and the glands tend to return to their normal adult form, thus emphasising the relative importance of the general host factor. Nevertheless these cells still differ from normal adult cells in that their responsiveness to testosterone and oestrone is greatly reduced. This may be due to deficiencies in the medium since if prostate glands from old mice are transplanted to young adult hosts the tissue may still show a secretory response. (Franks and Chesterman, not yet published).

One further point of interest is that the regenerative changes after injury in the first week of culture did not differ greatly in degree or kind in young, adult or old mice. A similar finding has been reported in a study of growth potential in regenerating rat livers (Glinos and Bartlett, 1951).

### SUMMARY

Oestrone, oestradiol monobenzoate and testosterone propionate had a direct action on organ cultures of mouse ventral prostate, oestrogens causing epithelial atrophy and testosterone stimulation. The extent of these changes may be affected by other hormones present in a medium containing human serum and chicken plasma.

Tissues from young mice ($1\frac{1}{2}$ months) were more sensitive to the depressant effects of oestrone and less sensitive to testosterone propionate.

Tissues from old mice (24 months) were relatively less responsive to both oestrone and testosterone propionate.

The most consistent response to both hormones was obtained with tissues from adult mice (4–5 months).

All cultures showed regenerative changes in the first week of culture, probably following trauma during dissection. This reaction was similar in degree and kind in tissues from young, adult and old mice.

The prostates from old mice before culture showed areas of irregular epithelial hyperplasia which regressed after culture on media containing normal adult serum.

The possible significance of these changes is discussed.

### REFERENCES

Andrew, W.—(1941) *Anat. Rec.*, **81**, 36.—(1944) *Amer. J. Anat.*, **74**, 97.
Emmens, C. W.—(1950) in ' Hormone Assay ', ed. Emmens, C. W., New York, (Academic Press), p. 405.
Fell, H. B. and Robison, R.—(1929) *Biochem. J.*, **23**, 767.
Franks, L. M.—(1954*a*) *J. Path. Bact.*, **68**, 617.—(1954*b*) *Ann. Roy. Coll. Surg. Engl.*, **15**, 236.—(1956) *Lancet*, ii, 1037.
Gerall, A. A.—(1958) *Endocrinology*, **63**, 280.
Glinos, A. D. and Bartlett, E. G.—(1951) *Cancer Res.*, **11**, 164.
Hamburger, C.—(1950) in ' Hormone Assay ', ed. Emmens, C. W., New York (Academic Press), p. 181.
Korenschevsky, V. and Paris, S. K.—(1950) *Cancer Res.*, **3**, 903.
*Iidem* and Benjamin, B.—(1950) *J. Geront.*, **5**, 120.
Lasnitzki, I.—(1951) *Brit. J. Cancer*, **5**, 345.—(1954) *Cancer Res.*, **14**, 632.—(1955*a*) *J. Endocrin.*, **12**, 236.—(1955*b*) *Brit. J. Cancer*, **9**, 434.
Mann, T., Davies, D. V. and Humphrey, G. R.—(1949) *J. Endocrin.*, **6**, 75.
Ortiz, E., Price, D., Williams-Ashman, H. G. and Banks, J.—(1956) *Endocrinology*, **59**, 479.
Preedy, J. R. K. and Aitken, E. H.—(1957) *Lancet* i, 191.
Segaloff, A., Flores, A. and Steelman, S. L.—(1955) *J. clin. Endocrin.*, **15**, 847.
Seaman, A.—(1956) *Exp. Cell Res.*, **11**, 283.
Shaffer, B. M.—(1956) *Ibid.*, **11**, 244.
Trentin, J. J.—(1950) *Cancer Res.*, **10**, 582.